# A deep investigation into the adipogenesis mechanism: Profile of microRNAs regulating adipogenesis by modulating the canonical Wnt/β-catenin signaling pathway

**DOI:** 10.1186/1471-2164-11-320

**Published:** 2010-05-23

**Authors:** Limei Qin, Yaosheng Chen, Yuna Niu, Weiquan Chen, Qiwei Wang, Shuqi Xiao, Anning Li, Ying Xie, Jing Li, Xiao Zhao, Zuyong He, Delin Mo

**Affiliations:** 1 State Key Laboratory of Biocontrol, School of Life Science, Sun Yat-Sen University, Guangzhou, 510006, China

## Abstract

**Background:**

MicroRNAs (miRNAs) are a large class of tiny non-coding RNAs (~22-24 nt) that regulate diverse biological processes at the posttranscriptional level by controlling mRNA stability or translation. As a molecular switch, the canonical Wnt/β-catenin signaling pathway should be suppressed during the adipogenesis; However, activation of this pathway leads to the inhibition of lipid depots formation. The aim of our studies was to identify miRNAs that might be involved in adipogenesis by modulating WNT signaling pathway. Here we established two types of cell model, activation and repression of WNT signaling, and investigated the expression profile of microRNAs using microarray assay.

**Results:**

The high throughput microarray data revealed 18 miRNAs that might promote adipogenesis by repressing WNT signaling: miR-210, miR-148a, miR-194, miR-322 etc. Meanwhile, we also identified 29 miRNAs that might have negative effect on adipogenesis by activating WNT signaling: miR-344, miR-27 and miR-181 etc. The targets of these miRNAs were also analysed by bioinformatics. To validate the predicted targets and the potential functions of these identified miRNAs, the mimics of miR-210 were transfected into 3T3-L1 cells and enlarged cells with distinct lipid droplets were observed; Meanwhile, transfection with the inhibitor of miR-210 could markedly decrease differentiation-specific factors at the transcription level, which suggested the specific role of miR-210 in promoting adipogenesis. Tcf7l2, the predicted target of miR-210, is a transcription factor triggering the downstream responsive genes of WNT signaling, was blocked at transcription level. Furthermore, the activity of luciferase reporter bearing Tcf7l2 mRNA 3' UTR was decreased after co-transfection with miR-210 in HEK-293FT cells. Last but not least, the protein expression level of β-catenin was increased in the lithium (LiCl) treated 3T3-L1 cells after transfection with miR-210. These findings suggested that miR-210 could promote adipogenesis by repressing WNT signaling through targeting Tcf7l2.

**Conclusions:**

The results suggest the presence of miRNAs in two cell models, providing insights into WNT pathway-specific miRNAs that can be further characterized for their potential roles in adipogenesis. To our knowledge, present study represents the first attempt to unveil the profile of miRNAs involed in adipogenesis by modulating WNT signaling pathway, which contributed to deeper investigation of the mechanism of adipogenesis.

## Background

Adipogenesis is the development of fat cells from preadipocytes to mature adipocytes, and has been one of the most intense studied models of cellular differentiation [1], which implicated in insulin resistance, type 2 diabetes, hypertension and atherosclerosis, collectively called the metabolic syndrome (MS)[2]. Wingless-type MMTV integration site family (WNTs) is indispensable for the regulation of adipogenesis [3-5], which is a family of secreted glycoproteins with autocrine and paracrine effects on the regulation of cell proliferation, survival, fate and behavior [6,7]. In canonical WNT signaling, WNT members bind to frizzled (FZD) receptors and low-density lipoprotein receptor-related protein-5 or -6 (LRP5/6) co-receptors, leading to inactivation of the degradation complex that includes glycogen synthase kinase (GSK)-3β, axin and adenomatous polyposis coli (APC), in turn blocking β-catenin phosphorylation by GSK-3β. Hypophosphorylation of β-catenin and translocation into the nucleus leads to binding with members of the lymphoid-enhancer-binding factor/T-cell-specific transcription factor (LEF/TCF) family and activation of WNT target genes [8,9]. As a member of LEF/TCF family, transcription factor 7 like 2 (Tcf7l2, formerly called Tcf4) is an important transcription factor triggering the downstream responsive genes of WNT signaling [10]. Previous research has shown that WNT signaling represses adipocyte differentiation by blocking the expression of Pparg and Cebpa, two transcription factors indispensable for adipogenesis [5]. Wnt10b was first identified as an inhibitor of adipogenesis, which must be suppressed for pre-adipocytes to differentiate in vitro [5]. In vivo experiments also demonstrated that Wnt-10b transgenic mice showed a similar decline in total body fat as well as perirenal depots [11]. Ectopic expression of Wnt-1 in 3T3-L1 pre-adipocytes blocked adipogenesis [12]. Together, these results indicated that the Wnt/β-catenin signaling pathway suppresses adipocyte differentiation and is therefore a molecular switch of adipogenesis; once it is activated, pre-adipocytes are maintained in an undifferentiated state [8].

Recently, it has been found that RNAs of a new class of noncoding RNAs, microRNAs (miRNAs), regulate gene expression in metazoans from C. elegans to humans [13,14]. miRNAs are endogenous non-coding RNAs, 22-24 nucleotides in length, which are demonstrated to modulate diverse biological processes through negatively regulating gene expressions posttranscriptionally by controlling mRNA stability or translation[15]. Many studies have shown that miRNAs participate in multiple metabolic processes including energy homeostasis, sugar/lipid metabolism and cell differentiation. In the development of lipogenesis, miR-143 was first reported to be up-regulated in human pre-adipocytes [16,17] and in adult mice in vivo [18], suggesting that it was involved in adipocyte differentiation; miR-375 has also been reported to promote 3T3-L1 preadipocytes differentiation [19]. To sum up, these findings indicated that WNT signaling and miRNAs have regulatory roles in adipogenesis.

To investigate which miRNAs might modulate WNT signaling during adipogenesis, we screened out the pupative miRNAs using microarray assay. Here we used 3T3-L1 cells to establish two models by different treatments. One treatment was to induce differentiation with an MDI cocktail (methylxanthine, dexamethasone and insulin), suppressing WNT signaling. The other was to treat the cells with lithium (LiCl), which is an inhibitor of GSK-3β that further blocks the phosphorylation of β-catenin, resulting in activation of WNT signaling [5]. The different miRNA expression patterns of the two models were investigated by a mouse miRNA microarray, enabling us to screen out a number of miRNAs that might regulate adipogenesis by activating or repressing the WNT signaling pathway.

## Results

### Cell models construction

**Figure 1:**
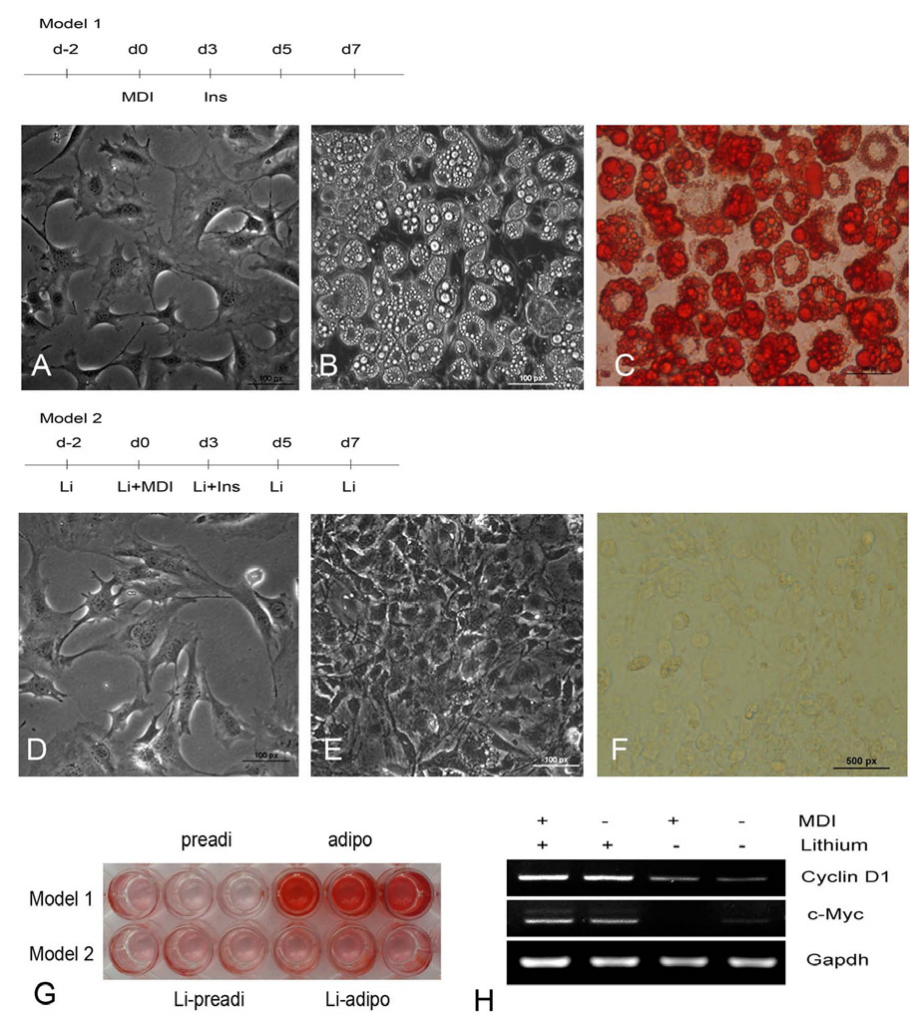
**Construction of two cell models**. (A) Pre-adipocytes; (B) Mature adipocytes differentiated for 7 days; (C) Oil red O staining of mature adipocytes; (D) Pre-adipocytes treated with lithium; (E) Induction of lithium-treated preadipocytes (Li+MDI) for 7 days; (F) Oil red staining of Li+MDI cells. (G) Low amplification of the two cell models. (H) Lithium activated the expression of Cyclin D1 and c-Myc at transcription level in 3T3-L1 cells.

**Figure 2:**
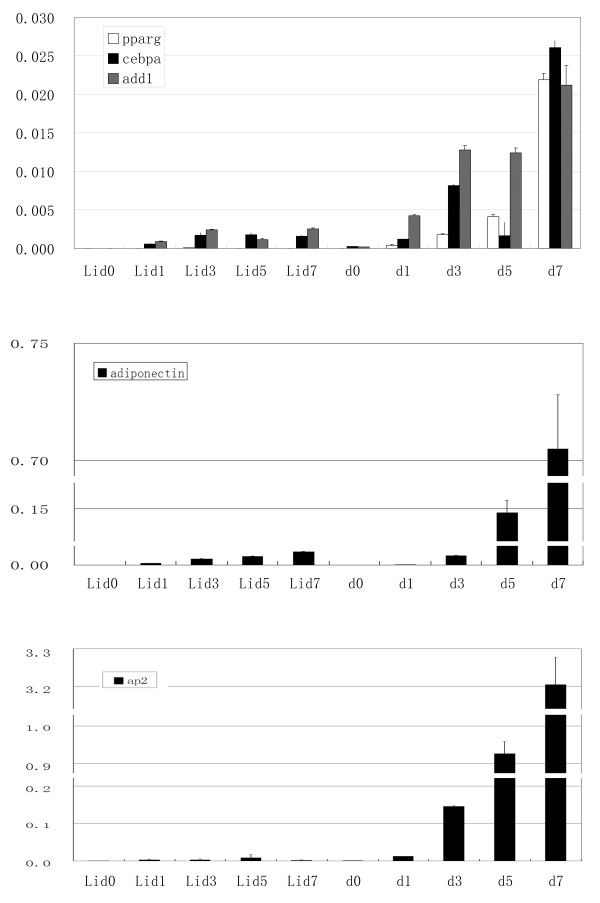
**The molecular evidence of cell models construction**. (A) Expression of Pparg, Cebpa, and Add1; (B) Adiponectin and (C) aP2 during induction in the two cell models. X-axis indicated the time course of differentiation. d0, d1, d3, d5, d7 mean the days of MDI induced differentiation; Lid0, Lid1, Lid3, Lid5, Lid7 mean the days of MDI induced differentiation of cells treated with Lithium. Y-axis indicates the expression of the detected genes relative to the internal reference gene *Gapdh*.

In model 1, pre-adipocytes (Figure 1, A, G) were induced to differentiate into mature adipocytes by MDI induction assay; enlarged cells with distinct lipid droplets were observed under the light microscope (Figure 1, B, C, G). In model 2, the pre-adipocytes treating with lithium before MDI induction (Figure 1, D, G) failed to differentiate into mature adipocytes (Figure 1, E, G), as confirmed by oil red O staining (Figure 1, F, G). As a control, NaCl was used in place of LiCl before MDI induction, the pre-adipocytes differentiated normally into mature adipocytes (Additional file 1). The following molecular data were fully consistent with these morphological findings. Target genes of the WNT signaling pathway such as *Cyclin D1 *and *c-Myc *were markedly more highly expressed in model 2 than model 1 cells (Figure 1, H). In time course experiments with model 1, several transcription factors such as Pparg, Cebpa and Add1 and differentiation-specific genes such as *aP2 *and *adiponectin *[20,21] increased significantly during adipogenesis. Interestingly, these genes were blocked completely in model 2 (Figure 2; Additional file 2). Both the morphological and molecular data showed that WNT signaling was dramatically suppressed and adipogenesis promoted in model 1, while in model 2, WNT signaling was activated and adipogenesis totally repressed. This suggested that the cell models had been constructed successfully.

### miRNA expression profile revealed by microarray data

#### (1) Expression profile of differentiation-specific miRNAs

To identify the miRNA related to adipocyte differentiation, miRNA expression in pre-adipocytes and adipocytes was analyzed by microarray. The results showed that 26 miRNAs underwent at least twofold changes, significant at P < 0.05 (Table 1, Additional file 3). Comparison between the two sets screened out most miRNAs; Nine were down-regulated and 17 up-regulated in the pre-adipocytes. Remarkably, miR-582-5p and miR-99a were up-regulated 6-fold and 5.24-fold respectively in mature adipocytes. Conversely, miR-196a and miR-130b were down-regulated 7- and 2.85-fold respectively in mature adipocytes. These were all significant differences (P < 0.01).

**Table 1:** Different expression of miRNAs between pre-adipocytes and mature adipocytes.

miRNA	P-value	Fold change	Regulation
mmu-miR-30e	0.00	2.30	up
mmu-miR-107	0.04	2.68	up
mmu-miR-468	0.05	3.03	up
mmu-miR-542-5p	0.02	4.38	up
mmu-let-7c	0.00	3.20	up
mmu-miR-582-5p	0.01	6.25	up
mmu-miR-335-5p	0.03	3.16	up
mmu-miR-10b	0.01	2.47	up
mmu-miR-101a	0.05	2.43	up
mmu-miR-99a	0.01	5.24	up
mmu-miR-101b	0.00	2.93	up
mmu-miR-146b	0.00	4.70	up
mmu-miR-210	0.03	2.41	up
mmu-miR-148a	0.00	2.99	up
mmu-miR-194	0.01	5.29	up
mmu-miR-103	0.02	3.70	up
mmu-miR-192	0.01	2.10	up
mmu-miR-29b	0.01	2.06	down
mmu-let-7e	0.03	2.16	down
mmu-miR-344	0.03	2.87	down
mmu-miR-196a	0.01	7.37	down
mmu-miR-140	0.00	2.03	down
mmu-miR-872	0.01	2.13	down
mmu-miR-130b	0.01	2.85	down
mmu-miR-140*	0.00	2.12	down
mmu-miR-34b-3p	0.01	2.00	down

Up-regulation means the expression of the miRNAs are higher in mature adipocytes than pre-adipocytes; Down-regulation is the reverse.

#### (2) Expression profile of WNT pathway-specific miRNAs

To investigate miRNA profiles associated with the WNT signaling activation, miRNA expression between the preadipocytes treating with or without lithium was analyzed by microarray. The results showed 15 miRNAs differ-expression for twofold, significant at P < 0.05 (Table 2, Additional file 4). Seven of these were up-regulated and eight down-regulated in lithium-treated pre-adipocytes. miR-322* was down-regulated 3.72-fold and miR-183* was up-regulated significantly (P < 0.01) changing 4-fold, in lithium-treated pre-adipocytes. Notably, miR-503 was down-regulated dramatically, nearly 8-fold (P < 0.05), in these cells.

**Table 2:** Different expression of miRNAs between pre-adipocytes and lithium-treated pre-adipocytes.

miRNA	P-value	Fold change	Regulation
mmu-miR-183*	0.00	4.16	up
mmu-miR-186	0.01	2.08	up
mmu-miR-582-5p	0.03	3.17	up
mmu-miR-344	0.01	2.47	up
mmu-miR-24-1*	0.04	2.69	up
mmu-miR-18a*	0.01	2.40	up
mmu-miR-7a*	0.04	3.58	up
mmu-miR-322	0.01	2.91	down
mmu-miR-542-5p	0.03	3.58	down
mmu-miR-351	0.03	2.32	down
mmu-miR-322*	0.04	3.72	down
mmu-miR-214	0.02	2.19	down
mmu-miR-196a	0.03	3.06	down
mmu-miR-146b	0.02	2.85	down
mmu-miR-503	0.02	7.96	down

Up-regulation means the expression of the miRNAs are higher in lithium-treated pre-adipocytes than pre-adipocytes; Down-regulation means the expression of the miRNAs are lower in lithium-treated pre-adipocytes than pre-adipocytes.

#### (3) Expression profile of WNT pathway-specific miRNAs by further induction in lithium-treated cells

To clarify further the pattern of miRNAs specific to WNT signaling, we reinvestigated the miRNAs that were differentially expressed between the MDI (Figure 1, B) and Li+MDI (Figure 1, E) groups. As observed by light microscopy, Li+MDI-treated cells did not differentiate into mature adipocytes (Figure 1, E, F), while the cells of the MDI group differentiated fully (Figure 1, B, C). Microarray analysis showed that 23 miRNAs differed at least two-fold between these treatments (Table 3, Additional file 5): Ten were down-regulated and 13 up-regulated in the Li+MDI group. In Li+MDI-treated cells, miR-24-1* and miR-301a were up-regulated 4.31- and 3.72-fold respectively, whereas miR-468 was down-regulated 3.42-fold; all those differences were significant (P < 0.01). Remarkably, miR-344 was up-regulated about 10-fold and miR-322 down-regulated about 6-fold in Li+MDI-treated cells.

**Table 3:** Different expression of miRNAs between MDI and Li+MDI cells.

miRNA	P-value	Fold change	Regulation
mmu-miR-27b	0.00	2.80	up
mmu-let-7e	0.04	2.22	up
mmu-miR-301a	0.00	3.73	up
mmu-miR-23b	0.01	3.29	up
mmu-miR-344	0.00	9.84	up
mmu-miR-320	0.04	2.05	up
mmu-miR-222	0.01	2.17	up
mmu-miR-24-1*	0.01	4.31	up
mmu-miR-181b	0.04	2.12	up
mmu-miR-181a-1*	0.02	2.47	up
mmu-miR-7a*	0.01	2.59	up
mmu-miR-714	0.02	2.55	up
mmu-miR-181d	0.04	4.25	up
mmu-miR-322	0.01	5.55	down
mmu-miR-450a-5p	0.04	2.65	down
mmu-miR-468	0.01	3.42	down
mmu-miR-542-5p	0.02	2.32	down
mmu-let-7c	0.01	2.31	down
mmu-miR-335-5p	0.03	3.07	down
mmu-let-7c-1*	0.05	2.26	down
mmu-miR-30a	0.01	2.36	down
mmu-miR-148a	0.01	2.04	down
mmu-miR-194	0.02	2.24	down

Up-regulation means the expression of the miRNAs are higher in Li+MDI cells than MDI cells; Down-regulation means the expression of the miRNAs are lower in Li+MDI cells than MDI cells.

### Validation of microarray by real time qRT-PCR

**Figure 3:**
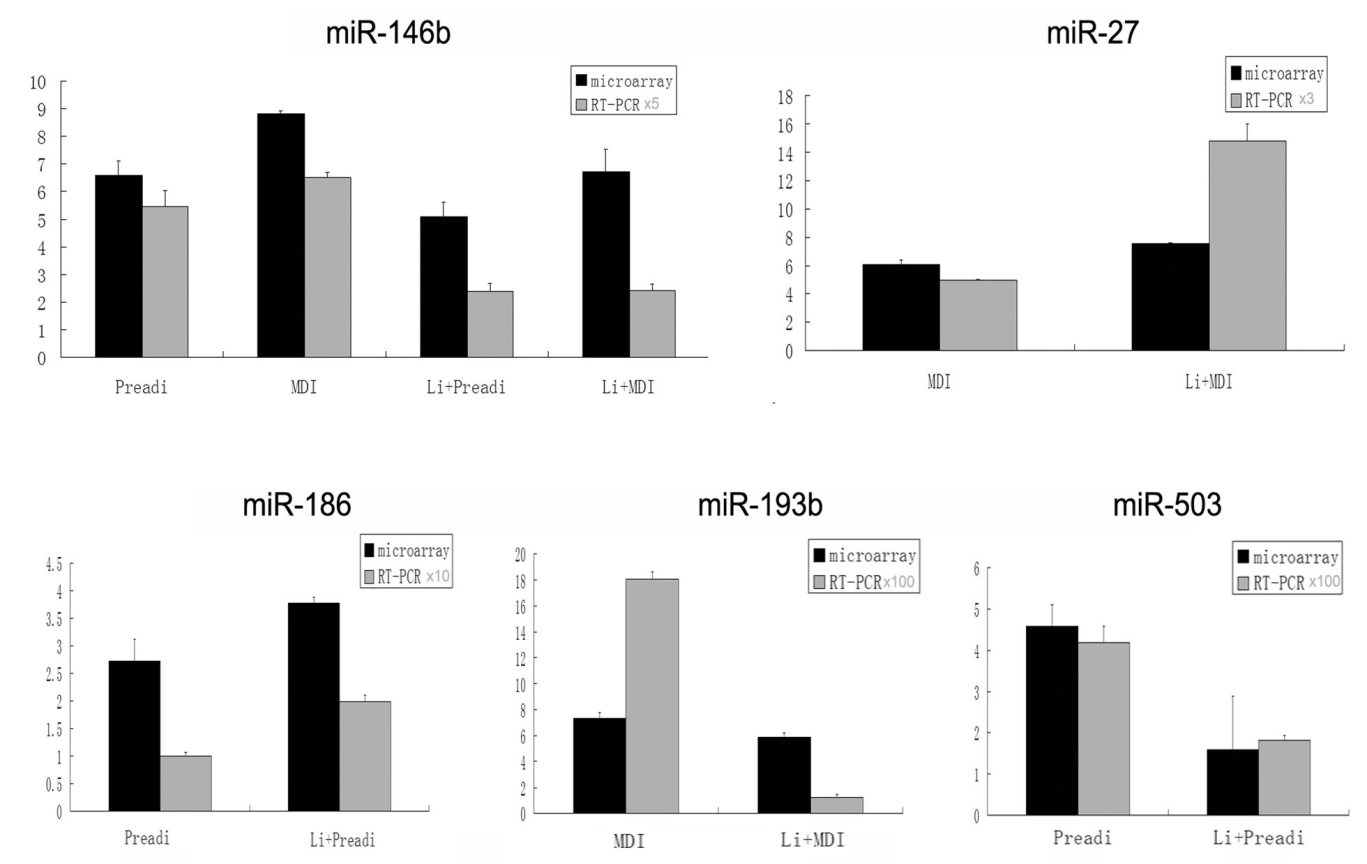
**Validation of the microarray results by qRT-PCR**. Preadip means pre-adipocytes, MDI means mature adipocytes, Li+Preadi means lithium-treated pre-adipoctyes, Li+MDI means the lithium treated 3T3-L1 cells followed with MDI induction remaining un-differentiation cells. Y-axis indicates the relative expression of the detected genes relative to the internal reference gene *Gapdh*; Fold amplifications are noted above.

To confirm the accuracy of the miRNA microarray, stem-loop polymerase chain reaction (qRT-PCR) assay was performed on several miRNAs chosen at random [22,23]. To determine whether there was a similar trend between the microarray and real time qPCR results, the latter were normalized by fold amplification. As shown in Figure 3, the qRT-PCR data (Additional file 2) mapped the microarray results closely. For example, the microarray data showed that miR-186 expression was higher in lithium-treated preadipocytes than in untreated preadipocytes, and the same trend was also observed in the qRT-PCR results. These indicated the precision and accuracy of the miRNA microarray used in the present study.

### Mir-210 promotes adipogenesis by repressing WNT signaling through targeting Tcf7l2

**Figure 4:**
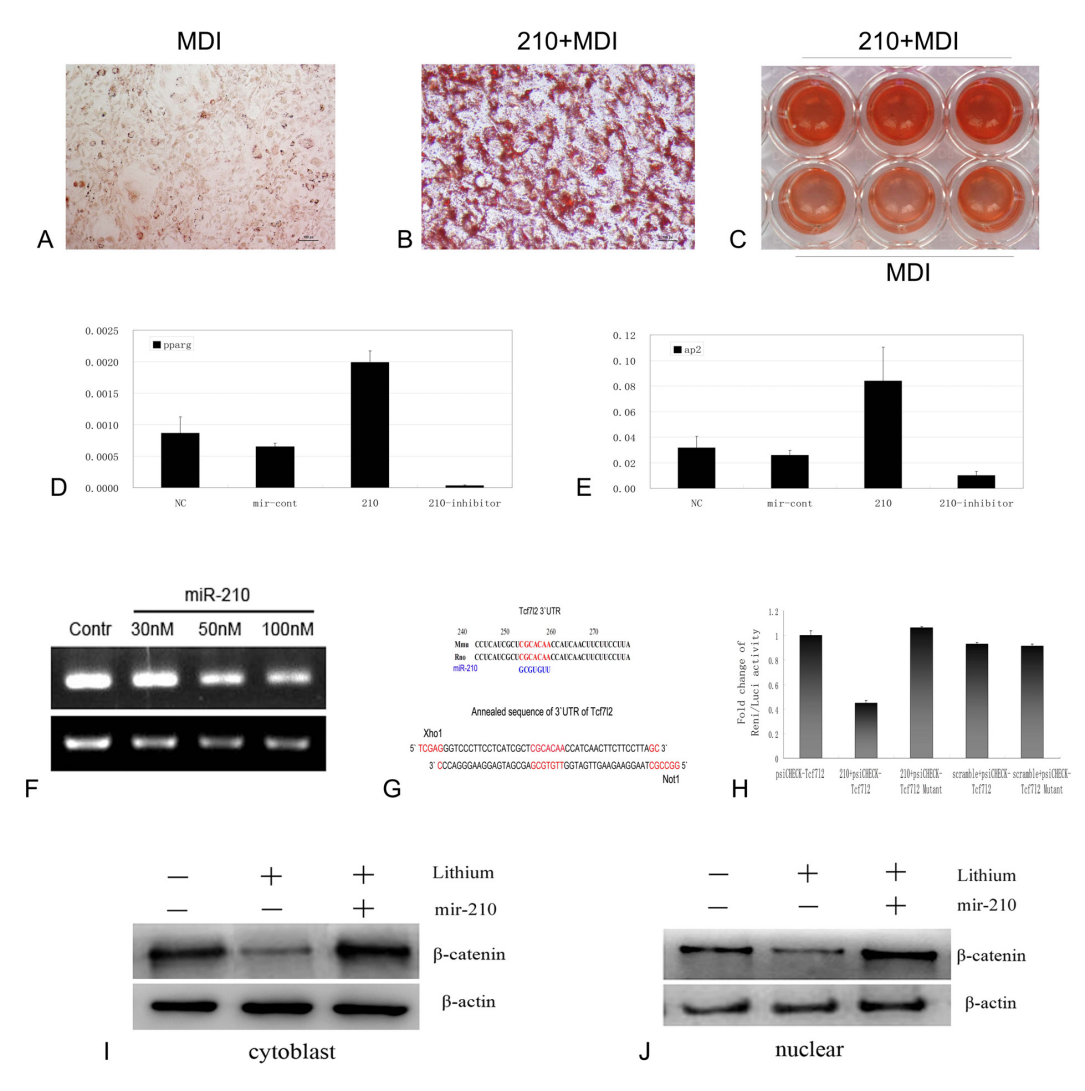
**Mir-210 represses WNT signaling through targeting Tcf7l2 and promotes adipogenesis**. (A) MDI induction for 3 days in 3T3-L1 cells; (B) Mir-210 mimics were transfected in 3T3-L1 cells and following 3 days' MDI induction; (C) Low amplification of 3T3-L1 cells with or without miR-210 transfection following 3 days' MDI induction; (D) Relative expression of Pparg and (E) aP2 after transfection of miR-210 mimics and inhibitor; (F) Mir-210 blocked the expression of Tcf7l2 at transcription level in dose-dependent manner in 3T3-L1 cells; (G) Mir-210 binding site in 3'UTR of Tcf7l2 (up) and the synthesized sequence of 3'UTR of Tcf7l2 with Xho1 and Not1 emzyme sites (down). (H) Mir-210 inhibits renilla luciferase reporter activity with 3' UTR binding site derived from Tcf7l2 in HEK293FT cells; (I) Transfection of miR-210 increased the protein of β-catenin in cytoloplasm and (J) nucleus in 3T3-L1 cells.

To validate the predicted targets and functions of these identified miRNAs, a mimic of miR-210 was transfected into 3T3-L1 cells for 24h before MDI induction, three days later, 100% of cell differentiated into mature adipocytes (Figure 4, B, C), while only less than 10% of the control cells differentiated (Figure 4, A, C). Meanwhile, qPCR data (Additional file 2) showed the differentiated specific factors such as Pparg and aP2 increased in transcription level after transfection with the mimics of miR-210, while both of the two factors decreased markedly after transfection with the inhibitor of miR-210 (Figure 4, D, E). The predicted target of miR-210, Tcf7l2 was repressed at the transcriptional level in a dose-dependent manner (Figure 4, F), and the activity of luciferase reporter containing the miR-210 binding site in the 3'UTR of Tcf7l2 mRNA (Figure 4, G) decreased for nearly 60% in HEK293FT cells (Figure 4, H). In lithium-treated 3T3-L1 cells, β-catenin protien was increased not only in the cytoplasm but also in the nucleus after transfection with miR-210 (Figure 4, I, J). These findings indicated that miR-210 promotes the lipids formation by repressing WNT signaling through targeting Tcf7l2.

## Discussion

For years, the mechanism and development of lipid formation have been actively investigated in the study of adipogenesis and obesity [2,3,24]. Many studies about the switch of adipogenesis, WNT signaling pathway, just focused on protein-coding genes such as *Wnt4*, *Wnt5 *and *Wnt10b *[8,24-26]. Recently, miRNAs have been found to have wide-ranging regulatory functions in cell proliferation, differentiation and many other processes [27], but only a few studies have focused on the interaction with WNT signaling during adipogenesis. Strikingly, one report showed that miRNAs have interaction with WNT signaling during the adipogenesis and miR-8 family members including miR-200a/b/c, miR-429 and miR-141 promoted adipogenesis [28]. Thus, miRNAs are believed to elucidate the mechanism of adipogenesis by regulating the Wnt/β-catenin signaling pathway [28]. In the current study, WNT-suppressed cells (Model 1), i.e. pre-adipocytes treated with a WNT-antagonist such as Insulin, differentiated into mature adipocytes (Figure 1, B, C, G). Meanwhile, the expression of adipocyte differentiation-specific genes such as *Pparg*, *Cebpa *etc. increased significantly (P < 0.01) after induction (Figure 2). These data indicated that the WNT-suppressed cell model was successfully established. In contrast, in the WNT-activated cell model (Model 2), i.e. preadipocytes treated with the WNT signaling activator lithium, adipogenesis was blocked completely (Figure 1, E, F, G) and the expression of adipogenesis-specific genes was totally suppressed (P < 0.01) (Figure 2), which suggests that the WNT-activated cell model was successfully established and the following experiments were reliable. Besides, combined investigation of WNT signaling pathway and miRNAs could offer a novel sight into the mechanism of adipogenesis.

**Figure 5:**
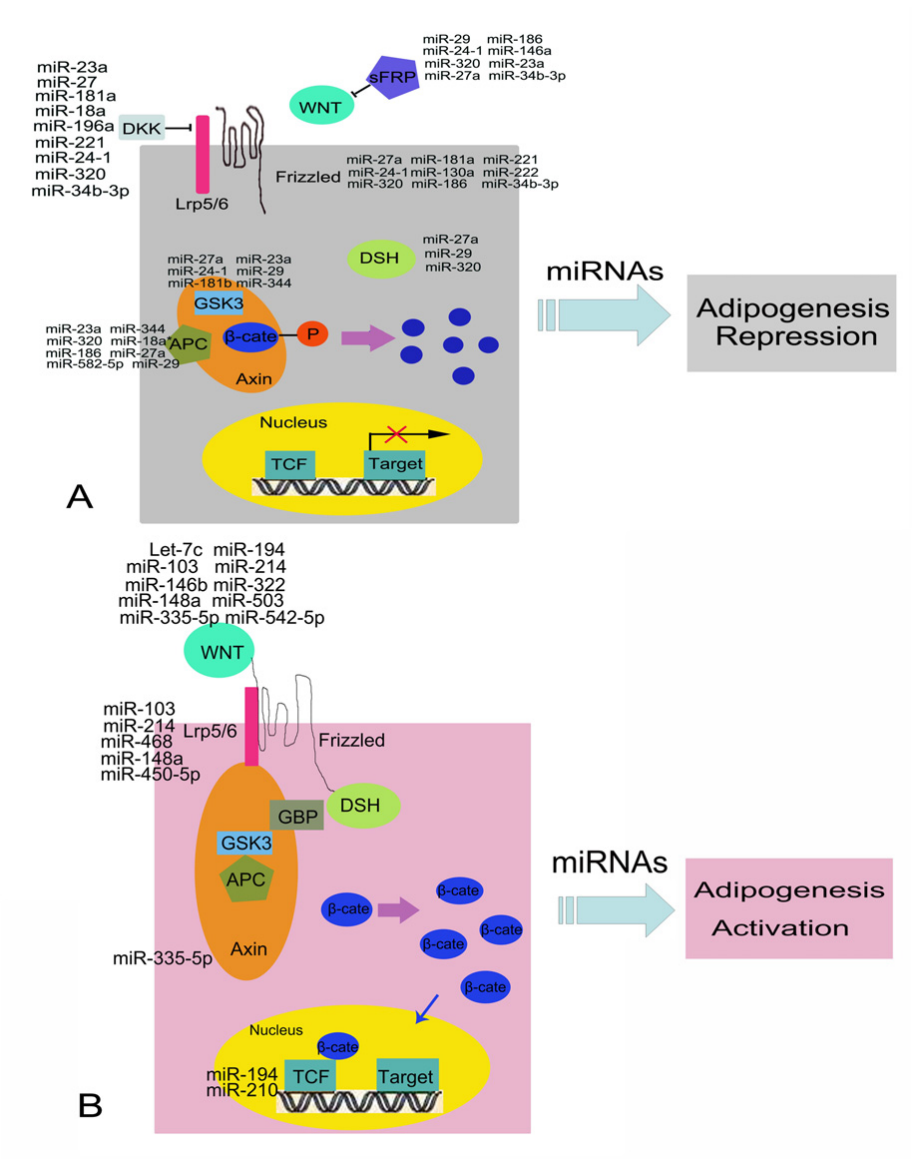
**The mode of miRNAs that regulate adipogenesis by modulating the canonical Wnt/β-catenin signaling pathway**. (A) Predicted miRNAs targets in WNT signaling pathway such as Sfrp, Dkk, Frizzled, Gsk3, Apc and Dsh, resulting in the repression of adipogenesis; (B) Predicted miRNAs targets of WNT signaling pathway such as Wnt, Lrp, Axin and Tcf, resulting in activation of adipogenesis. This figure was modified from a previous report [39].

According to the predicted potential function in adipogenesis by regulating WNT signaling, these miRNAs can be divided into two sets; one that suppresses WNT signaling and one that activates it. The former set should be characterized by up-regulation in mature adipocytes and down-regulation in both lithium-treated pre-adipocytes and cells of the Li+MDI group. These miRNAs also target the key factors in the WNT pathway, including members of WNT family such as Wnt10b, Wnt1 and Axin2, and molecules in the WNT pathway such as Lrp5/6, Tcf and Dvl (Additional file 6). The aforementioned screened pattern indicated that some miRNAs such as miR-103, miR-210, miR-148a, miR-320 and miR-194 accorded with this prediction. A previous report has shown that miR-103 was required for adipocyte differentiation, as confirmed by northern blotting [17]. In the present study, miR-103, which was markedly up-regulated in differentiating adipocytes (P < 0.05), was predicted to target Wnt3a and consequently to promote adipogenesis; Thus, our prediction was consistent with the previous study. The second set of miRNA (potentially activating Wnt/β-catenin signaling) is characterized by down-regulation in mature adipocytes and up-regulation in lithium-treated pre-adipocytes and adipocytes in the Li+MDI group. Importantly, these miRNAs mostly target important transcriptional factors or differentiation-specific marker genes during adipogenesis, such as *Pparg*, *Cebpa *etc. (Additional file 7). They included miR-301a, miR-130a/b, miR-27a/b, miR-18a, miR-320 and miR-181a/b/c/d, and were predicted to activate WNT signaling, resulting in the suppression of adipogenesis. A previous study showed that the miR-27 gene family was down-regulated during adipocytes differentiation. Over-expression of miR-27 specifically inhibited adipocytes formation and blocked the expression of Pparg and Cebpa as well as fat tissue in obese mice [29]. A similar result in the present study validated this previous report. As shown in table 3, miR-27b was up-regulated significantly by about 2.8-fold (P < 0.01) in Li+MDI group cells, indicating that it was less necessary for adipogenesis; Mir-27a/b was also predicted to target a master regulator in adipogenesis, i.e. Pparg, indicating that they might be antagonistic to adipogenesis, which was consistent with the previous report. To sum up: using microarray data, we screened out two sets of miRNAs that might play a role in suppressing or activating WNT signaling during adipogenesis. This predicted pattern was shown in figure 5, which offers a model for the study of adipogenesis related to miRNAs and WNT signaling.

To date, only a small number of predicted targets have been experimentally validated, so further investigation is needed to clarify the function of these miRNAs [30-32]. Microarray data showed that miR-210 was up-regulated in adipocytes, which suggested the potential role of miR-210 in promoting lipogenesis. After transfection of miR-210, the lipogenesis was dramatically promoted (Figure 4, B, C), but when antagonizing miR-210, the differentiation specific genes such as *Pparg *and *ap2 *were makedly suppressed, which suggested the specific role of miR-210 in promoting adipogenesis (Figure 4, D, E). Tcf7l2, a key transcription factor in activation by WNT signaling [33], is essential for the β-catenin/TCF complex downstream of WNT signaling. As predicted, miR-210 targets Tcf7l2 with hybrid free energy -30.3 kcal/mol. After transfection of miR-210, Tcf7l2 was markedly repressed at the transcriptional level (Figure 4, F) and the activity of luciferase reporter decreased markedly (Figure 4, H), which indicated that miR-210 promoted the lipid formation and Tcf7l2 should be targeted by miR-210. When WNT signaling was activated by lithium, β-catenin protein decreased dramatically. Interestingly, after transfection with miR-210, the β-catenin protein increased not only in the cytoplasm but also in the nucleus (Figure 4, I, J). These lines of evidence suggested that miR-210 probably plays a role in promoting the adipogenesis and repressing WNT signaling through targeting the transcription factor Tcf7l2. These experiments not only validated our hypothesis but also confirmed the accuracy and reliability of the predicted target of these miRNAs.

Mir-143, which was reported to be required during adipocyte differentiation [16], was not listed in our microarray data. In the present study, microRNAs that changed at least twofold and differed markedly (P < 0.05) between the two cell models were identified as potentially interesting. qRT-PCR showed that miR-143 expression in adipocytes after seven days' differentiation was 1.41 fold higher than in pre-adipocytes (data not shown). In a previous study, northern blotting indicated that miR-143 changed significantly on the 9th day after MDI induction, while the sample in the current work was harvested on the 7th day. As for the strict screening criterion, the expression of miR-143 does not change dramatically before the 9th day of differentiation and therefore was not shown in table 1[17].

The list of hybrids represents the free energies needed for micrRNAs to bind their targets: the lower the value, the more stable the binding, which offers an alternative way of assessing the reliability of predictions. To evaluate this screening criterion, we carried out experiments to find a reliable hybrid score by transfection of miRNA in vitro and conducting semi-quantitative PCR to detect the target genes. The results showed a good correlation in the miRNAs and predicted targets when the hybrid free energy was less than -30 kcal/mol (data not shown). Consistent with our observation, this criterion was validated by other reports using a similar approach [17]. For example, the hybrid free energy of miR-146a and its predicted target wnt5a was -30.3 kcal/mol; our prediction was confirmed by previous study. Therefore, hybrid free energy is recommended for consideration in the prediction of miRNA targets; the prediction is more reliable when hybrid score is less than -30 kcal/mol.

## Conclusions

In summary, the current work used microarray experiments to examine global miRNA expression in two 3T3-L1 cell models, i.e. activation or suppression of WNT signaling by MDI induction or lithium treatment. miRNA target prediction and further functional characterization of miR-210 will supplement the results of this study and help to elucidate the specific roles in adipogenesis. The findings demonstrated crosstalk between WNT signaling and miRNAs, highlighting the role of miRNAs during adipogenesis, i.e. repression and promotion of adipogenesis by regulating WNT signaling. The global changes in miRNA expression discovered in the present study may for the first time elucidate the preliminary mechanisms underlying WNT signaling-mediated changes in lipid formation during adipogenesis.

## Methods

### Cell culture and cell models construction

The 3T3-L1 (ATCC, CL-173TM) pre-adipocyte cell line was maintained in Dulbecco's modified Eagle's medium supplemented with 10% fetal bovine serum (Gibco, USA) at 37°C in 5% CO2-air. To construct a cell model in which WNT signaling was suppressed, 3T3-L1 cells were allowed to reach confluence and then exposed to MDI medium for three days; MDI contained 3-isobutyl-1-methylxanthine (0.5 M), dexamethasone (1 μM) and insulin (1.75 M). The cells were then cultured in high-glucose DMEM, 10% FBS and insulin alone for the following three days, and subsequently cultured with high-glucose DMEM and 10% FBS, replaced at 2-day intervals before the end of the checkpoint. To construct a cell model in which WNT signaling was activated, LiCl was added to the medium at a concentration of 25 mM. To determine whether WNT signaling was activated, the cells were further treated with MDI (Li+MDI treatment) to see whether they differentiated into mature adipocytes.

### RNA isolation and microarray experiment

Total RNA was extracted using a mirVana™ miRNA Isolation kit (Ambion, Inc., Austin, TX) according to the manufacturer's instructions. RNA quality was tested with Agilent Bioanalyzer 2100 (Agilent Technologies, Santa Clara, CA). Microarray hybridization was performed using an Agilent's miRNA microarray system (V12.0), covering 627 mouse miRNAs and 39 mouse viral miRNAs from miRBase database v 12 (Agilent Technologies, Foster City, CA) [34]. Total RNA was directly labeled using a Agilent's miRNA Complete Labeling and Hyb Kit (p/n 5190-0456) with Cy3. Hybridization was carried out at 65°C for 17 h and the arrays were then washed and scanned on the Agilent Scan Control software, then analyzed with Feature Extraction Software 9.5.3 (Agilent) using default parameters. Each sample was run in triplicate and the data were normalized using standard procedures [35].

### Microarray data analysis

Differentially expressed miRNAs were identified using t-tests with P-values < 0.05. The background-subtracted data were then subjected to variance stabilization normalization [36]. The data were further analyzed by clustering, and expression profiles were visualized with GeneSpring 10.0 (Agilent Technology).

### Validation of differentially expressed miRNAs by qRT-PCR

To validate the accuracy of the microarray, a stem-loop qRT-PCR assay based on SYBR Green I was performed on five miRNAs selected at random. The RT primers and real-time PCR primers were designed as described [22]. Real-time PCR was performed using a Roche Light Cycler 480 Real-Time PCR System. Briefly, 1 μg total RNA was reverse-transcribed for 5 min at 72°C, 5 min on ice, 60 min at 42°C, 5 min at 95°C. The 10 μl PCR reaction system contained 100 ng cDNA and 0.2 μl universal reverse and specific forward primers. The reaction protocol was 95°C for 5 min followed by 40 cycles at 95°C for 15 s, 60°C for 20 s and 72°C for 15 s. All reactions were run in triplicate. The expression of each miRNA relative to U6 RNA was determined by the 2^-Δ^CT method [37]. qPCR experiments were performed in triplicate and the data are presented as mean ± SD.

### Prediction and analysis of candidate miRNA targets

On line software packages were used to predict the genes targeted by miRNAs. We mainly used TargetScan [38]http://www.targetscan.org/mmu_50/ and hybrid http://bibiserv.techfak.uni-bielefeld.de/rnahybrid/submission.html.

### Transfection with microRNA mimics and inhibitors

3T3-L1 cells were plated 1 day before transfection. The duplex oligonucleotide (mimic) or single antisense of miR-210 (inhibitor) (Ribobio, China) was transfected into it using Lipofectamin 2000 (Invitrogen, USA) according to the manufacturer's instructions. After 24 h transfection, the cells were harvested for further experiments.

### Luciferase reporter assays

3'UTR segments containing the miR-210 binding site of Tcf7l2 were synthesized and annealed into double strands and inserted into the end of Renilla luciferase of the psiCHECK-2 vector (Promega, USA). HEK293FT cells were transiently transfected for 24 h with the psiCHECK-Tcf7l2, co-transfected with miR-210 mimics (10 ng) or a scrambled miR-control (10 ng) and psiCHECK-Tcf7l2 (50 ng) or psiCHECK-Tcf7l2 mutant plasmid (50 ng, mutation in the seed region in the 3'UTR of Tcf7l2), Data were presented as normalized ratios of target (Renilla) luciferase activity to control (Firefly) luciferase activity. Each transfected well was assayed in triplicate.

### Western blotting

Cytoplasmic and nuclear extracts were harvested with a nuclear-protein kit (Biyotime, China), fractionated by 12% SDS polyacrylamide gel electrophoresis, electroblotted on to a PVDF membrane using an iBlot™ Dry Blotting System (Invitrogen, USA), and then blocked with TBST containing 5% nonfat milk for 1 h at room temperature and incubated overnight at 4°C in a solution containing 2.0 μg/mL mouse anti-mouse β-catenin (R&D, USA). The membrane was then incubated at room temperature for 1 h in a solution containing a 1:2,000 dilution of HRP-conjugated sheep anti-mouse IgG, and the chemiluminescence was detected using ECL equipment (ImageQuant RT ECL, GE, USA).

## Authors' contributions

LQ performed research and drafted the manuscript. YC coordinated the study. YN contributed to qPCR Assays for miRNAs. WC and QW participated in the western blotting experiment. SX and YX participated in acquisition, analysis and interpretation of the microarray data. JL and AL participated in cell culture and luciferase reporter assays. XZ and ZH participated in the critical revision and approval of the final manuscript. DM conceived the experimental design and corrected the manuscript. All authors read and approved the final manuscript.

## Supplementary Material

Additional file 1 Oil red staining of 3T3-L1 cells when replacing LiCl by NaCl before 7 days'MDI induction. Click here for file

Additional file 2 The data of qRT-PCR Click here for file

Additional file 3 Bicluster of microRNAs expression in pre-adipocytes and mature adipocytes. Click here for file

Additional file 4 Bicluster of microRNAs expression in pre-adipocytes and lithium-treated pre-adipocytes. Click here for file

Additional file 5 Bicluster of microRNAs expression in MDI cells and Li+MDI cells. Click here for file

Additional file 6 miRNAs targets that potentially repress WNT signaling during adipogenesis. Click here for file

Additional file 7 miRNAs targets that potentially activate WNT signaling during adipogenesis. Click here for file
