# Supplementary figures and images for: A deep investigation into the adipogenesis mechanism: Profile of microRNAs regulating adipogenesis by modulating the canonical Wnt/β-catenin signaling pathway
Source: BMC Genomics. 2010 May 23;11:320. doi: 10.1186/1471-2164-11-320 (PMC2895628; doi:10.1186/1471-2164-11-320)

**Oil red staining of 3T3-L1 cells when replacing LiCl by NaCl before MDI induction for 7 days.**

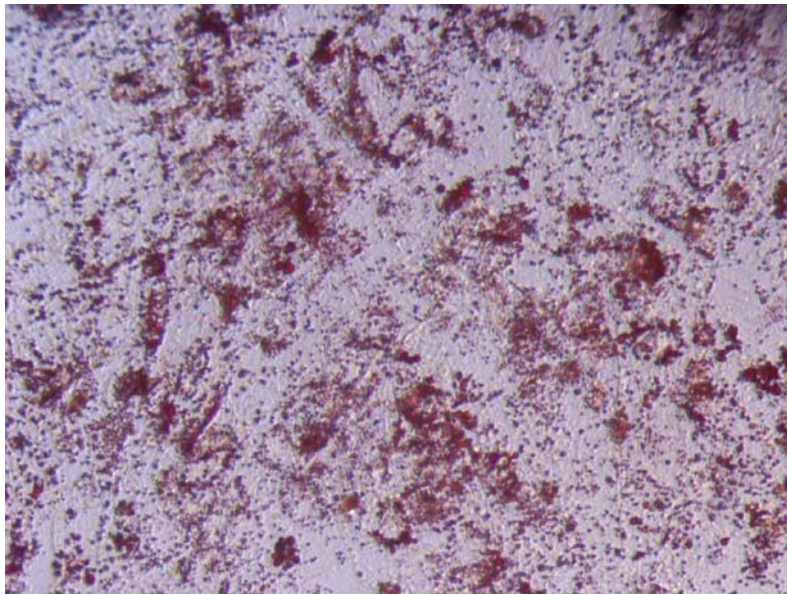

Supplement: Additional file 1 — Oil red staining of 3T3-L1 cells when replacing LiCl by NaCl before 7 days'MDI induction. [file 1471-2164-11-320-S1.PDF]

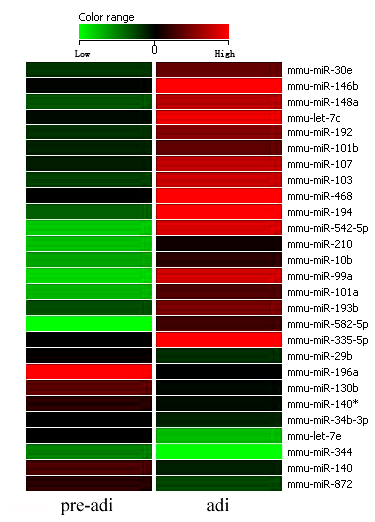

Supplement: Additional file 3 — Bicluster of microRNAs expression in pre-adipocytes and mature adipocytes. [file 1471-2164-11-320-S3.PNG]

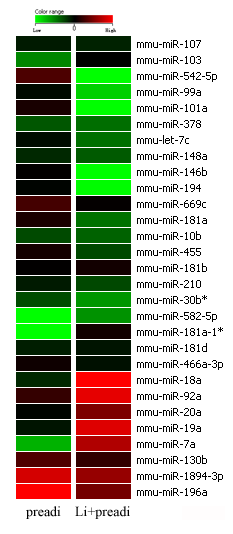

Supplement: Additional file 4 — Bicluster of microRNAs expression in pre-adipocytes and lithium-treated pre-adipocytes. [file 1471-2164-11-320-S4.PNG]

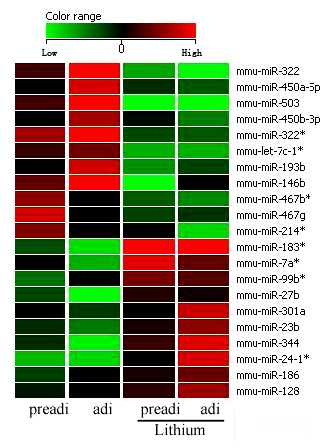

Supplement: Additional file 5 — Bicluster of microRNAs expression in MDI cells and Li+MDI cells. [file 1471-2164-11-320-S5.PNG]
