# Supplementary material for: A deep investigation into the adipogenesis mechanism: Profile of microRNAs regulating adipogenesis by modulating the canonical Wnt/β-catenin signaling pathway
Source: BMC Genomics. 2010 May 23;11:320. doi: 10.1186/1471-2164-11-320 (PMC2895628; doi:10.1186/1471-2164-11-320)
Supplement: Additional file 2 — The data of qRT-PCR [file 1471-2164-11-320-S2.PDF]

EXPERIMENTAL DESIGN

1) The figure 2. Validation of the two models by quantitative real time PCR of marker genes involved in WNT signaling and adipogenesis.

Definition of experimental and control groups

Answer Experimental groups are the cDNA of cell treating with MDI induction with or without Lithium. And the control groups are the cDNA of pre-adipocytes treated with or without Lithium.

Number within each group

Answer Experimental groups are Lid1, Lid3, Lid5, Lid7, d1, d3, d5, d7, and the control groups are Lid0, d0.

2) The figure 3. Mir-210 represses WNT signaling through targeting Tcf7l2 and promotes adipogenesis

Answer Experimental groups are miR-210 (mimics) and inhibitors, control groups are NC and miR-control.

3) The figure 4.Validation of the result of microarray by qRT-PCR

Definition of experimental and control groups

Answer

sample	Experimental groups	control groups
Mir-146b	Preadi	MDI
	Li+MDI	Li+Preadi
Mir-27	Li+MDI	MDI
Mir-186	Li+Preadi	Preadi
Mir-193b	Li+MDI	MDI
Mir-503	Li+Preadi	Preadi

Number within each group

Answer

sample	Number within each group
Mir-146b	Preadi, MDI, Li+MDI, Li+Preadi
Mir-27	Li+MDI, MDI
Mir-186	Li+Preadi, Preadi
Mir-193b	Li+MDI, MDI
Mir-503	Li+Preadi, Preadi

SAMPLE

Description

Volume/mass of sample processed

Answer 10μL

Processing procedure

Answer samples are fresh cultured cells.

NUCLEIC ACID EXTRACTION

Procedure and/or instrumentation

Name of kit and details of any modifications

Answer Takara 2×SYBRpremixEXTaqTM Kit

Details of DNase or RNAse treatment

Contamination assessment (DNA or RNA)

Nucleic acid quantification

Instrument and method

Answer We erased the contamination of instruments by RNase Decontamination Solution----RNaseZap® (Ambion Company).

The quality of RNA was measured by Spectrophotometers and

Fluorospectrometers---Nano drop (Thermo Fisher Company), in addition, agarose electrophoresis of RNA was also performed.

REVERSE TRANSCRIPTION

Amount of RNA and reaction volume

Answer 1µg of RNA was used to transcript, and the reaction volume was 25µL.

Priming oligonucleotide (if using GSP) and concentration

Answer Priming oligonucleotide was oligodT, with the concentration was 10pM

Reverse transcriptase and concentration

Answer M-MLV Reverse transcriptase (Promega Company) with the concentration was 200 units per 20µL reaction volume.

Temperature and time

Answer RNA+OligodT

70℃ 5min, ice 5min

M-MLV 5X Reaction Buffer

dNTP 10Mm each

Recombinant RNasin® Ribonuclease Inhibitor

M-MLV transcriptase

Nuclease-Free Water

42℃ 1h, 95℃ 5min, keep in -20℃

DATA ANALYSIS

qPCR analysis program (source, version)

Answer LightCycler 480 (Roche Company)

Version: Release 1.5.0

Cq method determination

Answer Cq is the point at which amplified product is first visible in the data.

Outlier identification and disposition

Answer If one samples` Cq volume is far different from other replicated samples would be removed, in addition, Cqs⩾40 could be ignored.

Results of NTCs

Answer melting curve was rough.

Justification of number and choice of reference genes

Answer reference gene was *Gapdh*.

Description of normalisation method

Answer Log2.

Number and stage qPCR of technical replicates

Answer qPCR was replicated in triplication.

Repeatability (intra-assay variation)

Answer The value of error represents repeatability of each sample.

1) The figure 2. Validation of the two models by quantitative real time PCR of marker genes involved in WNT signaling and adipogenesis.

	pparg	error	cebpa	error	ap2	error	adiponectin	error	add1	error
Lid0	1.03E-07	2.35E-08	1.56E-05	2.58E-06	3.56E-04	1.46E-05	4.32E-05	1.89E-06	2.89E-05	1.95E-06
Lid1	7.71E-06	2.01E-07	5.45E-04	6.44E-05	2.55E-03	2.55E-03	6.38E-04	6.06E-05	8.68E-04	8.48E-05
Lid3	1.26E-04	2.15E-05	1.73E-03	2.81E-04	2.69E-03	2.69E-03	2.65E-03	1.59E-04	2.41E-03	4.61E-05
Lid5	0.95E-05	0.21E-06	1.77E-03	2.31E-04	8.58E-03	8.58E-03	3.54E-03	1.50E-04	1.13E-03	1.17E-04
Lid7	1.07E-05	7.59E-06	1.59E-03	1.23E-04	1.29E-03	1.29E-03	5.65E-03	1.74E-04	2.53E-03	1.49E-04
d0	1.23E-05	1.41E-06	2.51E-04	1.81E-05	0.89E-03	1.28E-04	2.13 E-05	5.12E-06	1.76E-04	2.80E-05
d1	3.79E-04	1.36E-04	1.18E-03	2.03E-05	1.27E-02	2.66E-04	1.86E-04	6.35E-06	4.21E-03	1.37E-04
d3	1.80E-03	1.15E-04	8.15E-03	1.94E-04	0.1456	2.40E-03	3.97E-03	6.60E-05	1.28E-02	5.71E-04
d5	4.11E-03	2.56E-04	1.67E-03	1.67E-03	0.9262	3.28E-02	0.1484	5.38E-03	1.24E-02	6.60E-04
d7	2.19E-02	8.52E-04	2.61E-02	8.81E-04	3.205	7.30E-02	0.7048	2.32E-02	2.12E-02	2.56E-03

2) The figure 3. Mir-210 represses WNT signaling through targeting Tcf7l2 and promotes adipogenesis--Relative expression of Pparg and Ap2 at transcription level after transfection of miR-210 mimcs and inhibitor

	pparg	error	ap2	error
NC	8.65E-04	2.59E-04	3.16E-02	9.14E-03
mir-cont	6.49E-04	5.71E-05	2.60E-02	3.69E-03
210	1.99E-03	1.83E-04	8.39E-02	2.65E-02
210-inhibitor	3.33E-05	1.21E-05	9.95E-03	3.28E-03

3) Validation of the microarray results by qRT-PCR.

miR-146b						
	microarray	err	RT	err	RT-PCR*5	err*5
Preadi	6.5907	0.51699	1.094	0.1126	5.47	0.563
MDI	8.8226	0.08511	1.303	3.50E-02	6.515	0.175
Li+Preadi	5.0773	0.51707	0.4763	5.83E-02	2.3815	0.2915
Li+MDI	6.7254	0.80196	0.4833	4.41E-02	2.4165	0.2205

	microarray	err	RT	err	RT-PCR*100	err*100
Preadi	4.5704	0.52067	4.19E-02	3.85E-03	4.19E+00	3.85E-01
Li+Preadi	1.578045	1.31207	1.82E-02	1.02E-03	1.82E+00	1.02E-01

miR-186						
	microarray	err	RT	err	RT-PCR*10	err*10
Preadi	2.7144	0.40165	9.97E-02	6.24E-03	9.97E-01	6.24E-02
Li+Preadi	3.7738	0.10809	0.1987	1.16E-02	1.99E+00	1.16E-01

miR-27						
	microarray	err	RT	err	RT-PCR*3	err*3
MDI	6.0695	0.30776	1.654	2.89E-02	4.962	8.67E-02
Li+MDI	7.5532	0.07926	4.926	0.4061	14.778	1.22E+00

miR-193b						
	microarray	err	RT	err	RT-PCR*100	err*100
MDI	7.3576	0.37837	0.1807	5.45E-03	18.07	5.45E-01
Li+MDI	5.8568	0.32562	1.24E-02	1.56E-03	1.24	1.56E-01

Statistical methods for result significance

Answer variance test.

Software (source, version)

Answer SPSS software, version: SPSS 11.5.
