# Supplementary material for: A deep investigation into the adipogenesis mechanism: Profile of microRNAs regulating adipogenesis by modulating the canonical Wnt/β-catenin signaling pathway
Source: BMC Genomics. 2010 May 23;11:320. doi: 10.1186/1471-2164-11-320 (PMC2895628; doi:10.1186/1471-2164-11-320)
Supplement: Additional file 6 — miRNAs targets that potentially repress WNT signaling during adipogenesis. [file 1471-2164-11-320-S6.PDF]

Additional file 4 – miRNA targets that potentially repress WNT signaling during adipogenesis.

Underlining denotes that the gene is conserved in different species.

microRNA	WNT-related	Adipocyte-related	Hybrid-mfe (kcal/mol)
Mmu-mir-146b	Wnt1/10b, lrp11		Wnt10b:-23.9<br>Wnt1:-27.3<br>Lrp11:-23.4
Mmu-mir-542-5p	Wnt9b, Axud1		Wnt9b:-30.4
Mmu-mir-148a	<u>Wnt1/10b</u><br>Wnt16/26/9b/10a,<br>Wisp1,lrp8	Cebpe	Wnt10a:-23.2<br>Wnt10b:-25.3<br>Wnt1:-23<br>Wnt9b:-22.5<br>Wnt16:-25.2<br>Lrp8:-27.3<br>Wisp1:-25.7
Mmu-mir-194	<u>Tcf712</u><br>Wnt5a/6/9a	Cebpe	Wnt5a:-27.6<br>Wnt6:-24.7<br>Wnt9a:-28.7<br>Tcf712:-25.6
Mmu-mir-322	<u>Wnt3a/7a, wisp1,</u><br>Wnt2b/8a/11, wisp1		Wnt3a:-23.7<br>Wnt7a:-25.8<br>Wnt2b:-23.6<br>Wnt8a:-25.5<br>Wnt11:-30.1
Mmu-mir-335-5p	Axin1,Wisp2,<br>Wnt1/3/3a/5b/9b/10b		Wnt1:-15.5<br>Wnt3:-17.4<br>Wnt3a:-15.2<br>Wnt5b:-17.5

			Wnt9b:-17.7<br>Wnt10b:-20.6<br>Axin1:-17.5
Let-7c	Wnt1/9a/9b<br>Wnt3a/10a	Ppar-yc1a/b,<br>Cebpe, Ppar-a	Wnt1:-32.1<br>Wnt9a:-29<br>Wnt9b:-26.7
Let-7c-1*		Cebpe, Ppar-a	
Mmu-mir-103	Wnt3a, lrp1<br>Wnt7a/2/9b, Wisp1		Wnt3a:-29.5<br>Wnt7a:-30.2<br>Wnt2:-29.9<br>Wnt9b:-30.5
Mmu-mir-468	Lrp3/12		Lrp12:-29.1
Mmu-mir-214	Lrp8<br>Wnt2, Dvl1,<br>lrp2/4		Dvl1:-39.5<br>Wnt2:-30.4<br>Lrp8:-33.7<br>Lrp2:-33.9<br>Lrp4:-29.5
Mmu-mir-503	Wnt3a/7a		Wnt3a:-28.2<br>Wnt7a:-30
Mmu-mir-450a-5p	Lrp8		
Mmu-mir-30a		Ppar-y1a/b	Dkk1:-25.2
