# Supplementary material for: A deep investigation into the adipogenesis mechanism: Profile of microRNAs regulating adipogenesis by modulating the canonical Wnt/β-catenin signaling pathway
Source: BMC Genomics. 2010 May 23;11:320. doi: 10.1186/1471-2164-11-320 (PMC2895628; doi:10.1186/1471-2164-11-320)
Supplement: Additional file 7 — miRNAs targets that potentially activate WNT signaling during adipogenesis. [file 1471-2164-11-320-S7.PDF]

Additional file 5–miRNA targets that potentially activate WNT signaling during adipogenesis.

Underlining denotes that the gene is conserved in different species.

microRNA	WNT-related	Adipocyte-related	Hybrid- mfe (kcal/mol)
Mmu-mir-344	Apc, Gsk3b		Apc:-30.4<br>gsk3b:-30.3
Mmu-mir-24-1	Fzd5<br>Sfrp1, Dkk4, Gsk3b		Fzd5:-28.3
Let-7a*		Ppargc1a/b,<br>Cebpe, Ppara	Ppara:-24.6<br>Cebpe:-24.2
Let-7e		Ppargac1a/b,<br>Cebpe, Ppara	Ppara:-27.6<br>Cebpe:-27.1
Mmu-mir-196a	Igf2bp3, Nr6a1<br>Dkk1		Dkk1:-22.9
Mmu-mir-301a		Pparg	Pparg:-23.1
Mmu-mir-130a	Fzd4, Dkk4	Pparg	Pparg:-25.5
Mmu-mir-130b		Pparg	Pparg:-24.7
Mmu-mir-18a*	Apc2, Dkk3		Apc2:-29.6<br>Dkk3:-27.4
Mmu-mir-27b		Pparg	Pparg:-24.1
Mmu-mir-27a	Fzd7, Sfrp1, Apc, Dkk2<br>Wisp1, Fzd4/6/3/5,<br>Frzb, Dvl2, Gsk3b,<br>Gbp2/4/6	Pparg	Fzd7:-26.7<br>Pparg:-26.3<br>Gsk3b:-28
Mmu-mir-146a	Wisp1, Fzd7, Sfrp1		Fzd:-22.5<br>Wisp1:-25.1<br>Fzd7:-22.5

Mmu-mir-181a	Fzd5/10, Dkk1	Ppara	Sfrp1:-23.9<br>Dkk1:-26.2<br>Fzd5:-27.4<br>Fzd10:-27.7<br>Ppara:-27.1
Mmu-mir-181b	Wnt3a, Gsk3b	Ppara	Gsk3b:-28.6<br>Ppara:-26.7
Mmu-mir-181c		Ppara	Ppara:-27.8
Mmu-mir-181d	Wisp1	Ppara	Ppara:-25.2
Mmu-mir-582-5p	Fzd2, Apc<br>Sfrp1		Fzd2:-25.4<br>Apc:-23.8
Mmu-mir-29abc	Fzd5, Tcfap2c, Gsk3b<br>Wisp1, Fzd5/6, Sfrp4, Apc		Fzd5<br>:mir-29a/b:-25.1<br>Mir-29c:-22.6<br>Gsk3b<br>:mir-29a:-25.9<br>mir-29b:-24.2<br>mir-29c:-26.2
Mmu-mir-34b-3p	Fzd2/5, Sfrp1, Dkk2, Nkd2		Fzd2:-31.2<br>Fzd5:-27.4<br>Sfrp1:-26.7<br>Dkk2:-25<br>Nkd2:-23.2
Mmu-mir-186	Fzd10<br>Sfrp1/2, Fzd3/5, Apc/dd1		Fzd10:-24.5<br>Fzd3:-26.5<br>Fzd5:-27.5<br>Sfrp1:-24.7<br>Apc:-26.9
Mmu-mir-23b		Pparg, Fas	Pparg:-22.9

Mmu-mir-23a	Gsk3b, Grat2/1,<br>Gbp1/6<br>Sfrp1/5, Fzd5/7, Apc,<br>Dkk2	Gsk3b:-25<br>Frat1:-26<br>Frat2:-27.2<br>Sfrp1:-25.4<br>Sfrp5:-24.4<br>Fzd5:-27.9<br>Fzd7:-26.6<br>Apc:-25.3<br>Dkk2:-25.5
Mmu-mir-320	Wisp1,<br>Fzd7/1/4/10/5/6, Sfrp1,<br>Dvl2, Apcdd1, Dkk2/3,<br>Frat2	Wisp1:-31.2<br>Fzd7:-30.4<br>Dvl2:-32.6
Mmu-mir-222	Fzd5, Axin2	Fzd5:-31.3<br>Axin2:-28.3
Mmu-mir-221	Dkk4<br>Wisp1, Fzd4	Dkk4:-24.8<br>Wisp1:-26.2<br>Fzd4:-32.2
Mmu-mir-714	Camk2b	Camk2b :-32.6
